# The albumin-to-alkaline phosphatase ratio as an independent predictor of future non-alcoholic fatty liver disease in a 5-year longitudinal cohort study of a non-obese Chinese population

**DOI:** 10.1186/s12944-021-01479-9

**Published:** 2021-05-16

**Authors:** Guotai Sheng, Nan Peng, Chong Hu, Ling Zhong, Mingchun Zhong, Yang Zou

**Affiliations:** 1 Cardiology Department, Jiangxi Provincial People’s Hospital Affiliated to Nanchang University, Nanchang, 330006 China; 2 Gastroenterology Department, Jiangxi Provincial People’s Hospital Affiliated to Nanchang University, Nanchang, 330006 China; 3 Pediatrics Department, Lishui People’s Hospital, Lishui, 323000 China

**Keywords:** Albumin-to-alkaline phosphatase ratio, AAPR, Non-obese, Risk factors, Non-alcoholic fatty liver disease, Longitudinal cohort study

## Abstract

**Background:**

The albumin-to-alkaline phosphatase ratio (AAPR) is a newly developed index of liver function, but its association in patients with non-alcoholic fatty liver disease (NAFLD) has not been established. The aim of this study was to investigate the association between the AAPR and NAFLD in a non-obese Chinese population.

**Methods:**

The study included 10,749 non-obese subjects without NAFLD at baseline and divided them into quintiles according to the AAPR. A Cox multiple regression model was used to examine the association between the AAPR and its quintiles and the incidence of NAFLD.

**Results:**

The average age of the study population was 43.65 ± 15.15 years old. During the 5-year follow-up, 1860 non-obese subjects had NAFLD events. In the Cox multiple regression model, after adjusting the model according to important risk factors, the AAPR and NAFLD risk were independently correlated, and with a gradual increase in the AAPR, the NAFLD risk decreased gradually (HR: 0.61, 95% CI: 0.47, 0.81; *P*-trend< 0.0001). Additionally, there were significant interactions between the AAPR and BMI, blood pressure and lipids (*P*-interaction < 0.05). Stratified analysis showed that the risk of AAPR-related NAFLD decreased in people with normal blood pressure and lipid levels, while the risk of AAPR-related NAFLD increased abnormally in people who were underweight.

**Conclusions:**

This longitudinal cohort study provides the first evidence that the AAPR is an independent predictor of future NAFLD events in non-obese people. For non-obese people with a low AAPR, especially those with BMI < 18.5 kg/m^2^, more attention should be given to the management of risk factors for NAFLD to prevent future NAFLD.

**Supplementary Information:**

The online version contains supplementary material available at 10.1186/s12944-021-01479-9.

## Background

Non-alcoholic fatty liver disease (NAFLD) is a widespread chronic liver disease without a history of heavy alcohol consumption. It covers the development process of chronic diseases from simple steatosis of the liver to more severe non-alcoholic steatohepatitis and liver cirrhosis [1, 2]. However, in recent years, increasing evidence has shown that the disease burden of NAFLD comes not only from liver disease but also from NAFLD-related cardiovascular disease, metabolic disease and kidney disease [2–5]. NAFLD is a multi-system disease that affects multiple organs of the body and metabolic regulatory pathways [6, 7].

NAFLD is generally thought to be caused by overweight and obesity [2, 8], and in the past, related studies were mainly conducted in obese people. However, in recent years, an increasing number of studies have focused on non-obese NAFLD [9–11]. In a recent meta-analysis of more than 2 million people in 24 countries, non-obese people accounted for 40.8% of NAFLD patients globally [12], and in Asia, this situation seems to be more common [13, 14]. Additionally, a growing body of research suggests that people with non-obese NAFLD appear to be more prone to metabolic syndrome and progress to severe liver disease at a significantly faster rate [15, 16]. Therefore, it may be important to identify non-obese people at risk of NAFLD as early as possible and to manage their metabolic status.

Monitoring liver function markers, blood glucose and lipid metabolic markers and abdominal ultrasound are the most commonly used methods to assess the risk of NAFLD [17]. Albumin (ALB) and alkaline phosphatase (ALP) are the main indexes often used to evaluate liver function in clinical practice, in which the level of ALB can reflect the protein synthesis ability of the liver; ALP is a hydrolytic enzyme widely distributed in various tissues of the human body. It is mainly concentrated in the liver. When liver injury occurs, the level of ALP in the circulation increases [18]. Recently, in a study of liver tumour disease comparing the effects of different liver function measures on long-term prognosis, it was found that the albumin-to-alkaline phosphatase ratio (AAPR) showed the highest C-index compared to other liver function measures [19]. This result has also been verified in some similar studies [20, 21]. On the other hand, in the early stage of NAFLD, ALB and routine liver enzymes are usually normal [22], which makes it difficult for clinicians to identify groups at high risk of NAFLD based only on liver function tests. Therefore, the purpose of this study was to identify a population at high risk of NAFLD as early as possible with the help of some commonly used clinical liver function markers. At present, the link between the AAPR and NAFLD has not been established. Therefore, based on a large-sample longitudinal non-obese cohort, the following hypotheses are proposed in this study: can the AAPR be used to predict future NAFLD events in the non-obese Chinese population?

## Methods

### Study design

The longitudinal cohort data of this study come from the Dryad database, which is open and free, allowing researchers to use database services freely according to the purpose of the study. According to the terms of service of the database, the data sources were quoted and marked in this study [23]. The packet provides data on 16,173 non-obese subjects without NAFLD, liver disease, diabetes, history of heavy drinking and baseline medication use recruited by Wenzhou People’s Hospital from Jan 2010 to Dec 2014. The study scheme was approved by the institutional review boards of Wenzhou People’s Hospital, informed consent was obtained from the subjects, and a 5-year follow-up was completed. The detailed study design has been mentioned in previous studies [24]. In this study, a secondary analysis was carried out based on the NAFLD longitudinal cohort, and the following were some design elements: study exposure factors: AAPR; outcome: new-onset NAFLD events; subjects: 10,749 non-obese subjects were analysed after excluding the subjects with ALP and ALB deletions.

### Data collection

As mentioned earlier [24], baseline clinical data such as age, sex, height, weight, and blood pressure were recorded using a uniform health questionnaire; blood pressure was measured in a sitting position in a quiet environment using a standard electronic sphygmomanometer, and systolic and diastolic blood pressures (S/DBP) were recorded. Body mass index (BMI) was calculated as height divided by weight squared. The measurement of biochemical indexes was tested by automatic analytical instruments (Abbott AxSYM) through standard methods. The biochemical parameters included in this study were as follows: ALP, ALB, blood urea nitrogen (BUN), aspartate aminotransferase (AST), creatinine (Cr), triglyceride (TG), uric acid (UA), total protein (TP), total cholesterol (TC), direct bilirubin (DBIL), fasting plasma glucose (FPG), gamma glutamyl transferase (GGT), globulin (GLB), low-density lipoprotein cholesterol (LDL-C), alanine aminotransferase (ALT), total bilirubin (TB), and high-density lipoprotein cholesterol (HDL-C).

### Diagnosis of NAFLD

Subjects were assessed for NAFLD by abdominal ultrasound once a year during follow-up. The diagnosis of NAFLD was based on the diagnostic guidelines issued by the Chinese Liver Disease Association in 2010 [25]. The main contents of the evaluation include (a) diffuse high echo of the liver relative to the kidney and spleen; (b) echo attenuation of deep liver; (c) liver mildly to moderately enlarged, margin rounded obtuse; (d) liver blood flow signal is weakened; and (e) the right lobe and diaphragm are obscured or only partially shown. The diagnostic criteria for NAFLD needed to meet the echo characteristics of the above item (a) plus any one of the other items.

### Statistical analysis

All statistical analyses in this study were conducted on Empower Stats (R, version 2.20) and statistical software R language (version 3.4.3), and a *P-*value of < 0.05 (2-tailed) was considered to indicate statistical significance. The main steps were divided into the following three steps:

Step one: The baseline characteristics of all patients were stratified according to the AAPR quintile, and the continuous variables were expressed as the mean (standard deviation) or median (interquartile range). One-way ANOVA or the Kruskal-Wallis H test was used for inter-group comparisons. The qualitative data were summarized as frequencies or percentages, and the chi-square test was used to check the differences between groups.

Step two: In the population diagnosed with NAFLD, linear regression was used to check the correlation between the AAPR and baseline data (Supplementary Table 1, Additional file 1). The variables significantly related to the AAPR may be auxiliary factors of the association between the AAPR and NAFLD and were included in the model as important adjustment variables in Cox multiple regression analysis [26]. Additionally, before establishing the multiple regression model, the collinearity between variables was checked, and the variance inflation factor (VIF) of each variable was calculated (Supplementary Table 2, Additional file 1). The variables with VIF > 5 were regarded as collinear variables and could not be included in the multiple regression model [27].

Step three: The incidence of NAFLD in the five AAPR groups was estimated by the Kaplan-Meier curve, and the comparison between groups was made by the log-rank test. To explore the association between the AAPR and NAFLD, a Cox multiple regression model was constructed, and the AAPR was input into the model to calculate the hazard ratio (HR) and 95% confidence interval (CI) of NAFLD caused by each 1-unit increase [28]. Five models were used, with the crude model being unadjusted. Model 1 adjusted for the clinical baseline index (age, sex, height, BMI and SBP). Model 2 adjusted for model 1 plus liver function markers (GGT, ALT, AST, GLB, and TP). Since the AAPR is the ratio of ALB to ALP, in order to avoid the potential confounding effect between the AAPR and these two variables, ALB and ALP were not included in model 2. Model 3 adjusted for model 2 plus the blood glucose metabolism marker FPG and kidney function marker Cr. Model 4 adjusted for model 3 plus lipid metabolic markers (TG, HDL-C, and LDL-C). Additionally, considering that the correlation between the AAPR and NAFLD may be different under different conditions [4, 5, 11], the researchers also conducted an exploratory hierarchical analysis in some subgroups and checked the differences between different hierarchical groups by the likelihood ratio test to determine whether there was an interaction.

## Results

### Characteristics of the subject

Among the 16,173 patients enrolled in the study, 10,749 non-obese subjects fulfilled the inclusion criteria for the present post hoc analysis. The baseline mean age was 43.65 ± 15.15 years, with slightly more male subjects than female subjects (54.90% vs 45.10%). Table 1 summarizes the baseline characteristics grouped by AAPR quintiles. In the group with a low AAPR, there were more males than females, and with an increase in the AAPR, the number of males decreased gradually, while the number of females increased gradually. In the group with a higher AAPR, the average BMI, weight, age, TC, AST, ALP, TP, GLB, BUN, LDL-C, GGT, Cr, UA, ALT, FPG, TG, SBP and DBP of the subjects were lower than those in subjects with a lower AAPR. In contrast, ALB and HDL-C levels were higher in the groups with higher AAPR values (all *P* < 0.05).

**Table 1:** Characteristics of the subject

AAPR	Q1(≥0.05, ≤0.5)	Q2(> 0.5, ≤0.59)	Q3(> 0.59, ≤0.69)	Q4(> 0.69, ≤0.81)	Q5(> 0.81)	*P*-value
N (%)	2150	2150	2148	2150	2151	
Sex						< 0.001
Women	919 (42.74%)	930 (43.26%)	968 (45.07%)	974 (45.30%)	1057 (49.14%)	
Men	1231 (57.26%)	1220 (56.74%)	1180 (54.93%)	1176 (54.70%)	1094 (50.86%)	
NAFLD						< 0.001
No	1640 (76.28%)	1713 (79.67%)	1772 (82.50%)	1860 (86.51%)	1904 (88.52%)	
Yes	510 (23.72%)	437 (20.33%)	376 (17.50%)	290 (13.49%)	247 (11.48%)	
Age, years	44.00 (33.00–56.00)	41.00 (32.00–53.00)	40.00 (31.00–51.00)	40.00 (32.00–52.00)	38.00 (30.00–50.00)	< 0.001
ALP, U/L	104.39 (26.44)	80.66 (6.10)	70.04 (4.96)	60.56 (4.51)	47.12 (7.12)	< 0.001
GGT, U/L	26.00 (20.00–42.00)	23.50 (18.00–34.00)	22.00 (17.00–31.00)	20.00 (16.00–28.00)	18.00 (14.00–25.00)	< 0.001
ALT, U/L	19.00 (14.00–27.00)	18.00 (13.00–24.00)	16.00 (12.00–23.00)	15.00 (12.00–21.00)	14.00 (11.00–19.00)	< 0.001
AST, U/L	23.00 (20.00–28.00)	22.00 (19.00–26.00)	22.00 (19.00–25.00)	21.00 (18.00–24.00)	20.00 (17.00–23.00)	< 0.001
TP, g/L	73.96 (4.66)	73.78 (4.22)	73.89 (4.13)	74.01 (4.10)	73.63 (4.13)	0.024
ALB, g/L	43.70 (41.70–45.60)	44.60 (42.60–46.20)	44.80 (42.90–46.50)	45.00 (43.30–46.70)	45.00 (43.30–46.80)	< 0.001
GLB, g/L	30.15 (27.52–33.00)	29.20 (26.80–31.67)	29.00 (26.70–31.50)	29.00 (26.60–31.40)	28.40 (26.10–30.90)	< 0.001
TB, μmol/L	11.00 (8.80–14.30)	11.40 (9.00–14.55)	11.80 (9.20–14.80)	11.70 (9.30–15.00)	11.30 (8.80–14.50)	< 0.001
DBIL, μmol/L	2.00 (1.40–2.70)	2.00 (1.50–2.70)	2.10 (1.50–2.80)	2.10 (1.50–2.90)	2.00 (1.50–2.70)	0.003
BUN	4.52 (3.80–5.50)	4.60 (3.80–5.50)	4.46 (3.70–5.30)	4.40 (3.64–5.30)	4.30 (3.50–5.20)	< 0.001
Cr, μmol/L	88.00 (75.25–99.00)	86.00 (74.00–96.00)	84.00 (72.00–95.00)	79.00 (68.00–93.00)	75.00 (66.00–90.00)	< 0.001
UA, mmol/L	312.07 (86.01)	308.58 (84.48)	303.51 (88.49)	288.76 (89.70)	268.70 (91.41)	< 0.001
FPG, mmol/L	5.15 (4.84–5.55)	5.08 (4.80–5.44)	5.07 (4.80–5.41)	5.05 (4.79–5.33)	4.99 (4.76–5.29)	< 0.001
TC, mmol/L	4.67 (0.80)	4.63 (0.73)	4.61 (0.75)	4.62 (0.71)	4.56 (0.72)	< 0.001
TG, mmol/L	1.30 (0.97–1.79)	1.26 (0.93–1.76)	1.17 (0.88–1.65)	1.09 (0.81–1.54)	0.97 (0.74–1.33)	< 0.001
HDL-C, mmol/L	1.40 (0.35)	1.40 (0.35)	1.43 (0.36)	1.50 (0.37)	1.54 (0.36)	< 0.001
LDL-C, mmol/l	2.37 (2.01–2.70)	2.33 (1.98–2.66)	2.31 (1.96–2.64)	2.28 (1.93–2.61)	2.25 (1.91–2.58)	< 0.001
Height, m	1.66 (0.76)	1.67 (0.76)	1.67 (0.76)	1.66 (0.77)	1.65 (0.75)	< 0.001
Weight, kg	60.75 (8.13)	61.22 (8.02)	60.80 (8.51)	59.50 (8.52)	57.90 (8.62)	< 0.001
BMI, kg/m^2^	22.15 (20.61–23.48)	22.09 (20.69–23.45)	21.89 (20.32–23.33)	21.49 (19.93–23.04)	21.16 (19.66–22.84)	< 0.001
SBP, mmHg	128.56 (17.84)	125.25 (17.24)	123.16 (16.72)	120.42 (15.77)	117.03 (15.28)	< 0.001
DBP, mmHg	76.09 (10.62)	74.87 (10.28)	74.07 (10.20)	73.05 (10.16)	71.16 (9.83)	< 0.001

Values are n(%) or mean (standard deviation) or median (interquartile range); *Abbreviations*: *AAPR* Albumin-to-alkaline phosphatase ratio, *BMI* Body mass index, *BUN* Blood urea nitrogen, *Cr* Creatinine, *UA* Uric acid, *FPG* Fasting plasma glucose, *TC* Total cholesterol, *TG* Triglyceride, *HDL-C* High-density lipoprotein cholesterol, *LDL-C* Low-density lipoprotein cholesterol, *ALP* Alkaline phosphatase, *GGT* Gamma-glutamyl transferase, *ALT* Alanine aminotransferase, *AST* Aspartate aminotransferase, *TP* Total Protein, *ALB* Albumin, *GLB* Globulin, *TB* Total bilirubin, *DBIL* Direct bilirubin, *DBP* Diastolic blood pressure, *SBP* Systolic blood pressure

### Incidence of NAFLD

**Fig. 1:**
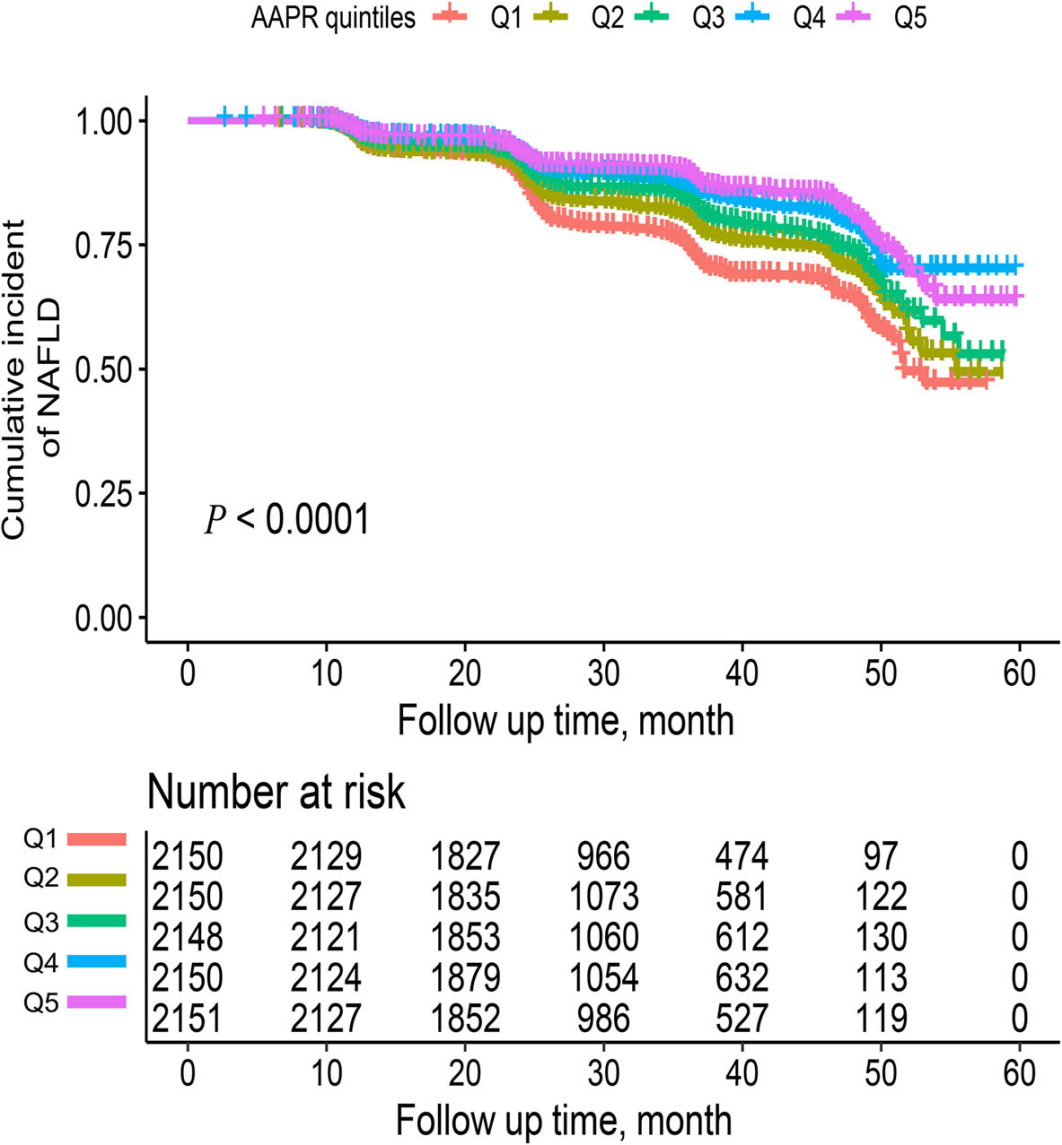
Kaplan-Meier curves compared the cumulative incidence of NAFLD at 5 years of follow-up after grouping by AAPR quintile (log-rank *P* < 0.0001). NAFLD: non-alcoholic fatty liver disease; AAPR: albumin-to-alkaline phosphatase ratio; Q1: Quintile 1; Q2: Quintile 2; Q3: Quintile 3; Q4: Quintile 4; Q5: Quintile 5

During the 5-year follow-up, 1860 non-obese subjects had NAFLD events. Among them, the NAFLD prevalence rates corresponding to the AAPR quintile grouping were Q1: 23.72%, Q2: 20.33%, Q3: 17.50%, Q4: 13.49%, and Q5: 11.48%. With the gradual increase in the AAPR, the incidence of NAFLD gradually decreased. In addition, the probability of 5-year cumulative NAFLD events in the five AAPR groups was estimated by the Kaplan-Meier curve to be Q1: 52.93%, Q2: 47%, Q3: 43.65%, Q4: 29.94% and Q5: 36.16% (Fig. 1). With the increase in the AAPR, the cumulative incidence of NAFLD decreased gradually (log-rank *P* < 0.001).

### Correlation analysis between the AAPR and baseline variables

Linear regression analysis showed that age, height, weight, SBP, ALP, ALB, GGT, ALT, AST, TP, GLB, Cr and FPG were associated with the AAPR in the population with NAFLD (*P* < 0.05). This finding suggests that these variables that were significantly related to AAPR may be auxiliary factors associated with AAPR and NAFLD.

### Association between the AAPR and NAFLD

To improve the model’s ability to identify the risk of NAFLD, the researchers established a Cox multiple regression model (Table 2). In the unadjusted model, there was a negative correlation between the AAPR and the risk of NAFLD, and the trend of NAFLD decreased with an increase in the AAPR (HR: 0.26, 95% CI: 0.20, 0.33; *P-*trend < 0.0001). After adjusting for the clinical baseline index (model 1), the negative correlation between the AAPR and NAFLD weakened, and the NAFLD risk corresponding to the AAPR quintile showed the same downward trend as before (HR: 0.41, 95% CI: 0.31, 0.53; *P-*trend < 0.0001). Then, after further adjustment for liver function markers in model 2, the association between the two was further reduced, and the negative correlation trend remained the same as before. Model 3 further adjusted for the blood glucose metabolism marker FPG and kidney function marker Cr, and the degree of negative correlation between the AAPR and NAFLD remained basically unchanged (HR: 0.54, 95% CI: 0.41, 0.72; *P*-trend< 0.0001). Finally, after further adjusting for the lipid metabolism markers (TG, HDL-C, and LDL-C) in the Cox multiple regression model, it was found that for each one-unit increase in the AAPR, the risk of NAFLD decreased by 39% (HR: 0.61, 95% CI: 0.47, 0.81, *P*-trend < 0.0001). Additionally, in the AAPR quintile groups, the group with the highest AAPR had a reduction in the NAFLD risk by 19% compared with the group with the lowest AAPR.

**Table 2:** Association between AAPR and NAFLD in different models

	HR (95%CI) *P*-value				
	Crude model	Model 1	Model 2	Model 3	Model 4
AAPR	0.26 (0.20, 0.33) < 0.0001	0.41 (0.31, 0.53) < 0.0001	0.52 (0.40, 0.69) < 0.0001	0.54 (0.41, 0.72) < 0.0001	0.61 (0.47, 0.81) 0.0004
AAPR (Quintile)					
Q1	Ref	Ref	Ref	Ref	Ref
Q2	0.80 (0.70, 0.91) 0.0005	0.81 (0.72, 0.93) 0.0017	0.89 (0.78, 1.02) 0.0874	0.90 (0.79, 1.02) 0.0999	0.91 (0.80, 1.04) 0.1490
Q3	0.67 (0.59, 0.77) < 0.0001	0.73 (0.64, 0.83) < 0.0001	0.82 (0.71, 0.94) 0.0045	0.83 (0.72, 0.95) 0.0075	0.85 (0.74, 0.98) 0.026
Q4	0.52 (0.45, 0.60) < 0.0001	0.61 (0.53, 0.71) < 0.0001	0.68 (0.58, 0.79) < 0.0001	0.66 (0.57, 0.77) < 0.0001	0.73 (0.63, 0.85) < 0.0001
Q5	0.47 (0.40, 0.55) < 0.0001	0.59 (0.84, 0.90) < 0.0001	0.67 (0.57, 0.78) < 0.0001	0.63 (0.53, 0.74) < 0.0001	0.72 (0.61, 0.85) < 0.0001
*P*-trend	< 0.0001	< 0.0001	< 0.0001	< 0.0001	< 0.0001

*Abbreviations*: *CI* Confidence interval, *HR* Hazard ratios, *AAPR* Albumin-to-alkaline phosphatase ratio

Crude model adjusted for none; model 1 adjusted for sex, age, height, BMI and SBP; model adjusted for model 1 plus liver function markers (GGT, ALT, AST, GLB, TP); model 3 adjusted for model 2 plus blood glucose metabolism marker FPG and kidney function marker Cr; model 4 adjusted model 3 plus lipid metabolic markers (TG, HDL-C, LDL-C)

### Subgroup analysis

In the exploratory subgroup analysis, the clinical baseline index data, kidney function index, lipid metabolic index, blood glucose metabolism index and liver function index were stratified according to the clinical cut-off points. The HR and 95% CI between different hierarchical groups were analysed and calculated by a Cox regression model, and the difference between hierarchical groups was checked by the likelihood ratio test to determine whether there was an interaction. As shown in Table 3, there was a significant interaction between factors such as BMI, SBP, and DBP in the association between the AAPR and NAFLD in the clinical baseline data subgroup (*P*-interaction< 0.05). Among them, the risk of AAPR-related NAFLD was abnormally increased in underweight people (BMI < 18.5 kg/m^2^, HR: 86.13, 95% CI: 5.86, 968.98; *P* = 0.0012), and in people with normal blood pressure (SBP < 140 mmHg, DBP < 90 mmHg), the risk of NAFLD associated with the AAPR was lower. In addition, significant interactions were observed in the lipid metabolism subgroup (*P*-interaction< 0.05) in which the risk of AAPR-related NAFLD decreased significantly when there was no abnormal increase in blood lipids. However, no significant interaction was observed in the subgroups of age, sex, liver function, kidney function and blood glucose metabolism.

**Table 3:** The effect size of AAPR on NAFLD in prespecified and exploratory subgroups in each subgroup

Characteristic	No. of participants	HR (95%CI)	*P*-value	*P*-interaction
Clinical baseline subgroup				
Age (years)				0.1504
< 30	2016	0.80 (0.40, 1.61)	0.5334	
≥ 30, < 45	4220	0.70 (0.47, 1.02)	0.0623	
≥ 45, < 60	2740	0.37 (0.22, 0.63)	0.0002	
≥ 60	1754	0.82 (0.42, 1.61)	0.5648	
Sex				0.9157
Men	5891	0.61 (0.43, 0.88)	0.0073	
Women	4839	0.63 (0.42, 0.94)	0.0252	
BMI, kg/m^2^				0.0076
≤ 18.5	821	86.13 (5.86, 968.98)	0.0012	
18.6–25	9909	0.60 (0.46, 0.79)	0.0003	
SBP, mmHg				0.0292
< 140	9076	0.54 (0.40, 0.74)	0.0001	
≥ 140	1657	0.94 (0.70, 1.28)	0.7140	
DBP, mmHg				0.0003
< 90	9857	0.48 (0.35, 0.65)	< 0.0001	
≥ 90	876	1.15 (0.89, 1.49)	0.2748	
Liver function subgroup				
GGT, U/L				0.8335
< 40	8977	0.63 (0.47, 0.84)	0.0016	
≥ 40	1770	0.67 (0.41, 1.08)	0.0996	
ALT, U/L				0.8653
< 40	10,109	0.61 (0.46, 0.81)	0.0008	
≥ 40	640	0.57 (0.24, 1.34)	0.1948	
AST, U/L				0.6524
< 40	10,419	0.69 (0.52, 0.90)	0.0063	
≥ 40	330	0.48 (0.10, 2.32)	0.8708	
Kidney function subgroup				
Cr, mmol/L				0.0247
< 108	9809	0.23 (0.17, 0.30)	< 0.0001	
≥ 104	937	1.37 (0.68, 2.76)	0.3732	
Blood glucose metabolism subgroup				
FPG, mmol/L				0.4721
< 6.1	9931	0.58 (0.43, 0.77)	0.0002	
≥ 6.1	799	0.76 (0.38, 1.55)	0.4578	
Lipid metabolism subgroup				
TC, mmol/L				0.0059
< 5.2	8372	0.48 (0.35, 0.67)	< 0.0001	
≥ 5.2	2358	0.93 (0.70, 1.23)	0.5994	
TG, mmol/L				0.0155
< 1.7	8348	0.48 (0.33, 0.68)	< 0.0001	
≥ 1.7	2382	0.88 (0.62, 1.25)	0.4658	
HDL-C, mmol/L				0.0340
< 0.9	392	2.01 (0.63, 6.38)	0.2368	
≥ 0.9	10,338	0.55 (0.45, 0.77)	< 0.0001	

The above model adjusted for model 4

Note: In each case, the model is not adjusted for the stratification variable

*Abbreviations*: *CI* Confidence, *HR* Hazard ratios;

## Discussion

To the best of our knowledge, this is the first report on the association between AAPR and new-onset NAFLD risk. In this study, after 5 years of follow-up, it was found that the increase in AAPR was negatively correlated with the risk of future NAFLD events in non-obese people. In the analysis of the Cox multiple regression model, the researchers determined that the AAPR was an independent predictor of NAFLD (HR: 0.61, 95% CI: 0.47, 0.81, *P*-trend < 0.0001).

The AAPR is the ratio of ALB to ALP, which can reflect some information regarding the two indicators at the same time, as well as information that cannot be reflected by these two indicators. In 2015, Chan et al. first reported that the AAPR can predict the poor prognosis of liver tumours, and its predictive performance is better than that of other liver markers [19]; some subsequent studies have also confirmed that this conclusion is reliable [20, 21]. At present, the AAPR has been used as a new liver marker to evaluate the long-term prognosis of liver tumour diseases. In this study, the researchers found that the AAPR can also be used to predict NAFLD in chronic liver diseases; the longitudinal cohort design of this study better reflects that the AAPR can independently predict early NAFLD risk. It is well known that ALB and liver function abnormalities are rarely seen in the early stage of NAFLD, so it may be difficult to detect potential NAFLD risks through conventional biochemical markers [22]. The findings of this study provide a new idea for the prevention of new-onset NAFLD.

In this study, the researchers also examined whether there were differences in AAPR-related NAFLD risk among people of different ages, sex, BMI, liver and kidney functions, blood pressure, blood glucose and blood lipids. The results showed that BMI, SBP, DBP, and lipid metabolism had significant interactions in the association between the AAPR and NAFLD (*P*-interaction < 0.05). Among those with normal blood pressure and lipids, the risk of NAFLD associated with the AAPR was reduced (SBP < 140 mmHg, DBP < 90 mmHg, TC < 5.2 mmol/l, TG < 1.7 mmol/l, HDL-C ≥ 0.9 mmol/l). However, the risk of AAPR-related NAFLD was abnormally increased in underweight individuals (BMI < 18.5 kg/m^2^, HR: 86.13, 95% CI: 5.86, 968.98; *P* = 0.0012), which may be related to the significant decrease in skeletal muscle mass in underweight individuals. Related studies have shown that with a decrease in BMI, the skeletal muscle weight, skeletal muscle index and body fat of the extremities decrease significantly [29], and low muscle mass is independently positively correlated with NAFLD [30]. Additionally, underweight people not only have an increased risk of NAFLD but also have a lower BMI, which often indicates malnutrition, which will significantly increase the incidence of adverse events [31, 32]. It is suggested that individuals with BMI < 18.5 kg/m^2^ should increase BMI to a normal level and improve skeletal muscle quality through diet and healthy exercise as soon as possible.

At present, there are very few studies on the AAPR, and the mechanism of the association between the AAPR and NAFLD is not clear. The results of this study were similar to those of previous studies. In this study, a low AAPR was an independent predictor of new-onset NAFLD events. It is generally believed that a low AAPR often indicates that ALB is too low or that ALP is too high. ALB is a very important protein in serum; it not only maintains the colloidal osmotic pressure of the body but also participates in the storage and as a conveyor of many substances [33]. The level of ALB reflects human nutritional status and liver function [18, 33]. In addition, ALB is also involved in the regulation of inflammation and the immune response [34, 35]. ALP is a hydrolytic enzyme found mainly in the liver, bone, intestine, kidney and placenta. ALP increases in those who are pregnant, suffer from bile duct disease, have impaired liver function or have bone disease [18, 36]. It has been reported that ALP is also related to the nutritional status of the body and has anti-inflammatory effects, which can inhibit the inflammatory response [37]. However, in this study, there were only 5 subjects whose ALB was toward the lower limit of the normal reference range, while only 53 people had ALP toward the upper limit of the normal reference range. In other words, the ALB and ALP levels of 99.49% of the population in this study were within the normal reference range, so malnutrition, inflammation and immune response do not seem like likely explanations for this association. A lower AAPR may affect the development of NAFLD in unique ways, the underlying mechanism of which is not clear, and further research is needed to explain this hypothesis in the future.

### Study strengths and shortcomings

This study has some unique advantages: (a) This is the first study to explore the association between the AAPR and NAFLD. The findings of this study provide a new idea for the prevention of new-onset NAFLD. (b) This study was a longitudinal cohort design with a large sample size. After strict statistical adjustment and sensitivity analysis, the negative correlation between the AAPR and NAFLD still stably existed, so the conclusion of this study can be considered relatively reliable. (c) The AAPR is the ratio of ALB to ALP, and the measurement of ALB and ALP is very simple and convenient in clinical practice, which is beneficial to the rapid application of the AAPR in clinical practice.

Of course, the shortcomings of this study are also obvious: (a) This study is the first to explore the association between the AAPR and NAFLD, so comparisons with similar studies and two-way verification of related basic research are lacking; therefore, the conclusions of this study should be carefully referred to, and more similar studies are needed to verify it. (b) This study is the second analysis of a previous study [24], and this study population was non-obese; considering that there are great differences between obese and non-obese people, more studies are needed to verify the correlation between the AAPR and NAFLD in obese people [10]. Additionally, although NAFLD-related variables have been widely collected in this study, there are still some variables that cannot be measured or obtained, which may lead to inevitable residual confusion. (c) In this study, the general clinical data and biochemical indicators of the subjects were standard parameters collected during physical examination, and repeated measurements were not carried out at the follow-up visits. Therefore, the impact of dynamic changes in baseline data on NAFLD could not be evaluated in this study. (d) In this study, NAFLD diagnosis was performed by ultrasound only, and no biopsy was conducted. Biopsy is the gold standard method to diagnose NAFLD stage [38]. Ultrasound has low sensitivity for the detection of mild steatosis [39], meaning that the subjects could already have steatosis but be classified as healthy liver. (e) The cohort of this study is made up of Chinese people, so the conclusion is only applicable to the Chinese population, while in other ethnic groups, the conclusion of this study is for reference only.

## Conclusions

In conclusion, this study demonstrated that a low AAPR is an independent predictor of NAFLD in the future. This finding provides new ideas for the prevention of new-onset NAFLD. Additionally, the AAPR is a new, simple, and inexpensive marker with a wide range of clinical application value.

## Supplementary Information

**Additional file 1: Supplementary Table 1.** Association between AAPR and baseline variables. **Supplementary Table 2.** Collinearity diagnostics steps.

## Data Availability

The data are available from the ‘Dryad’ database (www.datadryad.org).

## Abbreviations

AAPR: Albumin-to-alkaline phosphatase ratio
VIF: Variance inflation factor
LDL-C: Low-density lipoprotein cholesterol
NAFLD: Non-alcoholic fatty liver disease
ALB: Albumin
ALP: Alkaline phosphatase
Cr: Creatinine
BUN: Blood urea nitrogen
GGT: Gamma glutamyl transferase
CI: Confidence interval
S/DBP: Systolic and diastolic blood pressures
FPG: Fasting plasma glucose
BMI: Body mass index
AST: Aspartate aminotransferase
TG: Triglyceride
UA: Uric acid
TP: Total Protein
TC: Total cholesterol
DBIL: Direct bilirubin
HDL-C: High-density lipoprotein cholesterol
GLB: Globulin
ALT: Alanine aminotransferase
TB: Total bilirubin
HR: Hazard ratio
