# Supplementary material for: The albumin-to-alkaline phosphatase ratio as an independent predictor of future non-alcoholic fatty liver disease in a 5-year longitudinal cohort study of a non-obese Chinese population
Source: Lipids Health Dis. 2021 May 16;20:50. doi: 10.1186/s12944-021-01479-9 (PMC8126124; doi:10.1186/s12944-021-01479-9)
Supplement: Supplementary file 1 — Additional file 1: Supplementary Table 1. Association between AAPR and baseline variables. Supplementary Table 2. Collinearity diagnostics steps. [file 12944_2021_1479_MOESM1_ESM.docx]

Supplementary Table 1: Association between AAPR and baseline variables

|  | Statistics | Effect size (β) | *P*-value |
| --- | --- | --- | --- |
| Sex |  |  |  |
| Women | 819 (44.032%) | Ref |  |
| Men | 1041 (55.968%) | -0.009 (-0.033, 0.015) | 0.47713 |
| Age, years | 45.002 ± 15.112 | -0.001 (-0.002, -0.000) | 0.02238 |
| ALP, U/L | 78.156 ± 22.945 | -0.008 (-0.008, -0.007) | <0.00001 |
| GGT, U/L | 44.110 ± 43.997 | -0.001 (-0.001, -0.000) | <0.00001 |
| ALT, U/L | 25.860 ± 16.593 | -0.002 (-0.002, -0.001) | <0.00001 |
| AST, U/L | 25.048 ± 9.385 | -0.003 (-0.004, -0.001) | 0.00006 |
| TP, g/L | 74.040 ± 4.424 | 0.004 (0.001, 0.007) | 0.00534 |
| ALB, g/L | 44.592 ± 2.772 | 0.016 (0.012, 0.020) | <0.00001 |
| GLB, g/L | 29.448 ± 4.168 | -0.003 (-0.006, -0.000) | 0.04907 |
| TB, µmol/l | 12.416 ± 4.946 | 0.001 (-0.002, 0.003) | 0.68928 |
| DBIL, µmol/l | 2.009 ± 1.132 | 0.004 (-0.011, 0.019) | 0.58741 |
| BUN, mmol/l | 4.650 ± 1.305 | 0.002 (-0.007, 0.011) | 0.70888 |
| Cr, µmol/l | 88.104 ± 20.034 | -0.001 (-0.001, -0.000) | 0.01714 |
| UA | 331.842 ± 86.198 | 0.000 (-0.000, 0.000) | 0.92255 |
| FPG, mmol/l | 5.481 ± 1.022 | -0.013 (-0.025, -0.001) | 0.03036 |
| TC, mmol/l | 4.799 ± 0.791 | -0.007 (-0.022, 0.008) | 0.35962 |
| TG, mmol/l | 2.015 ± 1.491 | 0.007 (-0.001, 0.015) | 0.09524 |
| HDL-C, mmol/l | 1.295 ± 0.315 | 0.009 (-0.028, 0.047) | 0.62607 |
| LDL-C, mmol/l | 2.399 ± 0.457 | -0.012 (-0.038, 0.014) | 0.35061 |
| Height, m | 1.68 ± 0.074 | 0.002 (0.000, 0.004) | 0.01621 |
| Weight, kg | 65.305 ± 7.294 | 0.002 (0.001, 0.004) | 0.00788 |
| BMI, kg/m^2^ | 23.159 ± 1.348 | 0.006 (-0.003, 0.015) | 0.16212 |
| SBP, mmHg | 128.824 ± 16.040 | -0.001 (-0.002, -0.000) | 0.01431 |
| DBP, mmHg | 78.019 ± 10.149 | -0.000 (-0.002, 0.001) | 0.54527 |

Abbreviations: AAPR: Albumin-to-alkaline phosphatase ratio; BMI: body mass index; BUN: blood urea nitrogen; Cr: creatinine; UA: uric acid; FPG: fasting plasma glucose; TC: total cholesterol; TG: triglyceride; HDL-C: high-density lipoprotein cholesterol; LDL-C: low-density lipoprotein cholesterol; ALP: Alkaline phosphatase; GGT: gamma-glutamyl transferase; ALT: alanine aminotransferase; AST: aspartate aminotransferase; TP: Total Protein; ALB: albumin; GLB: globulin; TB: Total bilirubin; DBIL: Direct bilirubin; DBP: diastolic blood pressure; SBP: systolic blood pressure;

Supplementary Table 2: Collinearity diagnostics steps.

|  | Step 1 | Step 2 | Step 3 |
| --- | --- | --- | --- |
| AARC | 1.1 | 1.1 | 1.1 |
| Sex | 1.1 | 1.1 | 1.1 |
| Age | 1.1 | 1.1 | 1.1 |
| GGT | 1.3 | 1.3 | 1.3 |
| ALT | 3.5 | 3.5 | 3.5 |
| AST | 3.6 | 3.6 | 3.5 |
| TP | 3.2 | 3.2 | 3.2 |
| GLB | 3.3 | 3.3 | 3.3 |
| TB | 2 | 2 | 2 |
| DBIL | 2.2 | 2.2 | 2.2 |
| BUN | 1.6 | 1.6 | 1.6 |
| Cr | 1.6 | 1.6 | 1.6 |
| UA | 1.7 | 1.7 | 1.7 |
| FPG | 1.2 | 1.2 | 1.2 |
| TC | 6.6 | 6.6 | NA |
| TG | 2.3 | 2.3 | 1.5 |
| HDL | 2.7 | 2.7 | 1.4 |
| LDL | 4.9 | 4.9 | 1.1 |
| Height | 105.2 | 1.4 | 1.4 |
| Weight | 244.1 | NA | NA |
| BMI | 106.7 | 1.3 | 1.3 |
| SBP | 2.4 | 2.4 | 2.4 |
| DBP | 2 | 2 | 2 |

VIF = 1/(1-R^2^).

Table 3 Discrimination of the five Cox multiple regression models.

| Model diagnostic measure | Crude Model | Model I | Model II | Model III | Model IV |
| --- | --- | --- | --- | --- | --- |
| Discrimination |  |  |  |  |  |
| C index | 0.583 | 0.741 | 0.756 | 0.762 | 0.781 |

C index: concordance index.
